# For Colds

**Published:** 1898-04

**Authors:** 


### FOR COLDS.

We find in the *Therapeutische Monathepte* a remedy by Wunche for colds, which, he says, in the early stage acts almost as a specific. A mixture is prepared composed of from one to two parts of menthol and twenty parts of chloroform; of this, from four to six drops are placed in the hollow of the hand, the hands quickly rubbed together and placed over the face, inhaling deeply through mouth and nostrils. The application can be repeated two or three times during the day if necessary.—*The Med. Times.*

